# The Effectiveness of Conventional and Accelerated Methods of Orthodontic Traction and Alignment of Palatally Impacted Canines in Terms of Treatment Time, Velocity of Tooth Movement, Periodontal, and Patient-Reported Outcomes: A Systematic Review

**DOI:** 10.7759/cureus.24888

**Published:** 2022-05-10

**Authors:** Mahran Raheel Mousa, Mohammad Y Hajeer, Ahmad S. Burhan, Omar Heshmeh

**Affiliations:** 1 Department of Orthodontics, Faculty of Dentistry, Damascus University, Damascus, SYR; 2 Department of Oral and Maxillofacial Surgery, Faculty of Dentistry, Damascus University, Damascus, SYR

**Keywords:** periodontal status, acceleration of tooth movement, orthodontic traction, root resorption, pain and discomfort, piezosurgery, corticotomy, velocity of tooth movement, impacted canine traction, palatally impacted canine

## Abstract

The objective of the current review was to evaluate the effectiveness of traditional and accelerated methods of palatally impacted canine's (PIC) traction in terms of treatment duration, velocity, periodontal, and patient-reported variables.

An electronic search for randomized controlled trials (RCTs) and controlled clinical trials (CCTs) published between January 1990 and October 2021 was conducted in nine databases. Five major orthodontic journals were hand searched for additional studies. The participants were patients with unilateral or bilateral PICs who received conventional or accelerated orthodontic treatment with fixed appliances. Cochrane’s risk of bias tool (RoB 2 tool) for RCTs and ROBINS-I tool for CCTs were used to assess the risk of bias. The Grading of Recommendations Assessment, Development, and Evaluation (GRADE) guidelines were used to assess the overall quality of the evidence.

Nine articles (eight RCTs and one CCT) were included in this review (371 patients). There was no clarity in most studies about the possible effect of the mechanical traction method on treatment outcomes. The treatment duration decreased (about three to six months) when the open surgical method was used with traditional techniques and the traction velocity increased significantly (about 1-1.5 mm/month) when acceleration methods were used. No significant differences were found between the conventional intervention groups, as well as between the traditional and accelerated groups, in terms of most periodontal variables (p > 0.005).

No significant differences were found in the pain levels associated with traditional PICs' traction when comparing different exposure methods in the short-term follow-up (1-10 days), while contradictory results were found in the pain incidence between these methods. The relationship between the pain/discomfort levels and the type of mechanical traction method was not evaluated. According to the GRADE, the quality of evidence supporting these findings ranged from low to very low.

The combination of the open surgical technique with various designs of either superelastic wires or elastic traction means can lead to a reduction in the orthodontic treatment duration of PICs. The use of direct anchorage by miniscrews to move the PICs away from the adjacent teeth roots can lead to a reduction in root resorption and shorten the treatment duration. The evidence supporting these findings ranged from low to very low. The use of different types of mechanical means for conventional PICs' traction, with the use of open or closed traction techniques, does not lead to significant differences in periodontal outcomes between intervention groups. Contradictory results exist regarding the severity of the perceived pain in relation to the surgical exposure type, and the relationship between this variable and the mechanical traction method is still unclear. The use of accelerated methods for PICs' traction can lead to an increase in the velocity of traction movement with no significant differences in periodontal outcomes between accelerated and conventional methods. The evidence supporting these findings ranged from low to very low. More high-quality randomized CCTs are needed to establish good evidence in this field.

The protocol of this systematic review was registered in the International Prospective Register of Systematic Reviews (PROSPERO; CRD42021274476) during the first stages of this review.

## Introduction and background

Dental impaction is defined as the total or partial loss of tooth emergence that occurs after the normal age of tooth formation [1]. The impaction of upper canines remains one of the most frequently encountered surgical-orthodontic problems, with a reported incidence of 0.9-2.2% in the general population [2]. Ranked after the third molar impaction, the maxillary canine is the second most frequently impacted tooth with an incidence of 0.92-1.7%, most often with a palatal path of eruption. Palatal impactions were four times more frequent than vestibular impactions [3]. Upper impacted canines are a common problem and this is due to the length of the development period and the fact that the place of formation is the deepest within the bone relative to the other teeth in addition to the longest path of emergence between all teeth [1].

Common approaches for the management of impacted canines include early interceptive measures [4] or late intervention, including extraction [5] and surgical exposure of the canine's crown with a subsequent orthodontic alignment of the tooth [6]. Given the high aesthetic and functional value of canines, the combined surgical/orthodontic approach to relocate the impacted canine to its proper place in the dental arch is considered often, with two major surgical techniques: the open and the closed technique. As palatal gingiva is all attached, both closed and open surgical methods are appropriate [7].

Orthodontic guidance with surgical assistance is required after the accurate diagnosis of impaction and after ensuring that all-natural growth chance is exhausted and usually takes place at least six months after the completion of the apical root formation [8]. Advocates of the closed eruption approach note such benefits as the possibility to influence the direction of the extrusion of the impacted tooth, patient comfort during the healing process [9], reduced surgical bleeding, easier placement of the attaching device [10], and acceptable periodontal health after treatment [11].

The clinicians who support the open exposure technique and spontaneous eruption (also termed free eruption) of the canine claim several potential advantages: the ability to observe the impacted tooth movement during treatment, no need for attachment bonding, time-saving during the surgical procedure [12], fewer repeated operations necessary [13], and acceptable periodontal health after treatment [14]. Several mechanical methods were used for the eruption of impacted canines: the ballista loop [7], cantilever spring [15], K‑9 spring [16], and power chains [17]. The therapeutic efficacy of these methods is apparently not yet compared in any study.

Recently, acceleration of orthodontic movement has become of high interest [18], as surgical [19] and non-surgical [20] acceleration methods have become common in clinical practice. Some of the surgical methods were invasive [21,22] and some were minimally invasive [23,24].

The approach based on osteotomy of the cortical bone was considered helpful in stimulating the regional accelerated phenomena (RAP), which helps to activate the metabolic bone activities [25]. Corticotomy-assisted orthodontics has been used in many orthodontic procedures such as maxillary incisors retraction [26], upper canines retraction [27], and maxillary or mandibular crowded teeth alignment [28,29]. However, there appears to be a limited number of studies related to impacted canine movement acceleration in the medical literature. Palatally impacted canine (PIC) forced-eruption time averaged 6.6 months using the ostectomy-decortication technique compared with 21.0 months using open-closed surgical exposure techniques (i.e. 3.2 times more rapid) [30]. Fischer [31] reported six cases of bilateral PICs treated in a split-mouth design with randomly assigned surgical exposure on one side and selective alveolar decortication (without ostectomy) on the other; the treatment duration was 28-33% more rapid in the corticotomy-assisted technique.

To our knowledge, six systematic reviews have been performed to assess the differences in outcomes between open or closed surgical exposure of impacted canines [32-34]. These systematic reviews focused on the periodontal health of impacted canines following withdrawal, surgery time, post-surgical complications, patient's perceptions, the esthetic appearance of withdrawn and aligned impacted canines, and duration for canines' traction to the dental arch. In a recent systematic review by De Araujo et al. [33] about the surgical exposure techniques of PICs, they reported that the results of periodontal status and the surgical exposure duration were not different between the open or closed method. However, these systematic reviews have not focused on the orthodontic traction mechanism (type of appliance, methods of attachment, type of anchorage) or on the type of withdrawal technique (auxiliary springs, power chain, archwire auxiliaries). In a very recent systematic review by Guarnieri et al. [34], better post-treatment periodontal condition was found with the metallic-auxiliaries used for the force application system than the elastic auxiliaries. However, in that review, the auxiliaries were assessed based only on the type of material (elastic or metallic) and not on a specific type of auxiliary, which can affect the periodontal and dental results.

Moreover, the cumulative evidence related to the impact of these mechanical techniques regarding their overall comparative performance in terms of clinically relevant outcomes has not yet been objectively assessed. In addition, there have been no systematic reviews evaluating the adjunctive acceleratory procedures of PICs' traction.

Our review aims to systematically evaluate the recent literature in the assessment of the scientific evidence regarding the effectiveness of various treatment strategies of the PICs' traction and to critically assess whether differences exist between the traditional and the accelerated methods of withdrawing impacted canines in terms of speed and periodontal and patient-reported outcomes. Therefore, the question that the current review tries to answer is: What is the effectiveness of conventional and accelerated orthodontic fixed appliance treatment in withdrawing PICs in terms of treatment time, the velocity of tooth movement, and periodontal and patient-reported outcomes?

## Review

Protocol and registration

The absence of similar reviews was verified before writing and registering this review protocol by performing a scoping search in the PubMed database. A pre-registration of the protocol in the International Prospective Register of Systematic Reviews (PROSPERO) was done (CRD42021274476). This review was reported according to the Preferred Reporting Items for Systematic Reviews and Meta-Analyses (PRISMA) guidelines [35] and written according to the Cochrane Handbook [36].

Eligibility criteria

Inclusion and exclusion criteria were defined according to the following framework: participants, intervention, comparison, outcomes, and study design (PICOS).

Participants

Healthy patients with unilateral or bilateral PICs, both males and females between the ages of 12 and 35 years old, with any type of malocclusion, of any ethnic group, who received orthodontic treatment with fixed orthodontic appliances (without any predetermined restrictions on initial malocclusion or indication for treatment) were included.

Intervention

Alignment of a PIC using fixed orthodontic treatment with or without an acceleration procedure employing any type of mechanical traction.

Comparison

Spontaneous eruption of the contralateral canine or a conventional fixed orthodontic treatment using any type of mechanical traction (other than that used in the intervention group if the intervention group was not associated with an acceleration procedure).

Outcomes

The primary outcomes of this systematic review were the duration of orthodontic traction of impacted canines (the time from the start of the forced eruption until the canine reaches its final dental arch position), duration of complete orthodontic treatment, and the velocity of impacted canine movement. The secondary outcomes of this systematic review were periodontal outcomes (including gingival recession, pocket depth, and the width of keratinized tissues (KT)), patient-reported outcomes (including overall satisfaction, pain, and disruption of the function), and iatrogenic harm to teeth (canine or lateral incisor root resorption).

Study Design

Randomized controlled trials (RCTs), quasi-randomized controlled trials (Q-RCTs), and controlled clinical trials (CCTs) that were published between January 1990 and October 2021 in the English language were included.

Exclusion Criteria

Retrospective studies, in vitro studies, animal studies, finite element analysis studies, case reports or case series reports, editorials, personal opinions, reviews and technique description articles, articles without a reported sample, and studies with fewer than five patients in the experimental group were excluded. Also, studies that include labially impacted maxillary canines or mandibular impacted canines, studies that also include other impacted teeth than canines, non-English language trials, and studies with an absence of a control group or the presence of a control group of non-treated subjects (in two arms studies) were also excluded.

Search strategy

Articles were selected by conducting an electronic search of the literature published in the following computerized databases: CENTRAL, Embase®, Scopus®, MEDLINE, PubMed, Web of Science™, Google Scholar™, Trip, OpenGrey, and PQDT Open from Pro-Quest. The reference lists of selected papers and relevant reviews were screened for any possible related studies, which may have not been discovered by the electronic web-based search. ClinicalTrials.gov and the World Health Organization International Clinical Trials Registry Platform (ICTRP) were also checked electronically to retrieve any unpublished studies or currently accomplished research work. The terms used in the search were teeth*, tooth*, canine*, and cuspid*, in various combinations with impact*, ectopic*, transpose*, malposition*, eruption*, displace*, unerupted*, palatal*, and retain*. Details of the electronic search strategy are provided in Table 1.

**Table 1:** Electronic search strategy of the bibliographic databases

Database	Search strategy
CENTRAL (The Cochrane Library)	#1 orthodontic* OR "tooth movement" OR "orthodontic tooth movement" OR "tooth displacement " OR "orthodontic treatment" OR "orthodontic therapy". #2 (canine* OR cuspid) AND (impacted* OR retained* OR transposed* OR ectopia* OR eruption* OR displaced* OR malpositioned* OR unerupted*). #3 (impact*) AND (canine* OR cuspid* (AND (upper* OR maxillary* OR palatally* OR unilateral* OR bilateral*). #4 (impact*) AND (canines* OR cuspids*) AND (maxillary* OR palatally*) AND (treatment* OR exposure* OR technique* OR approach* OR eruption* OR method* OR management* OR traction* OR withdrawal*). #5 accelerat* OR rapid* OR short* OR speed* OR fast OR velocity OR duration OR rate OR time OR "regional accelerated phenomenon" OR RAP. #6 #3 OR #4 OR #5 #7 #1 AND #2 AND #6
Embase	#1 orthodontic* OR "tooth movement" OR "orthodontic tooth movement" OR "tooth displacement " OR "orthodontic treatment" OR "orthodontic therapy". #2 (canine* OR cuspid) AND (impacted* OR retained* OR transposed* OR ectopia* OR eruption* OR displaced* OR malpositioned* OR unerupted*). #3 (impact*) AND (canine* OR cuspid* (AND (upper* OR maxillary* OR palatally* OR unilateral* OR bilateral*). #4 (impact*) AND (canines* OR cuspids*) AND (maxillary* OR palatally*) AND (treatment* OR exposure* OR technique* OR approach* OR eruption* OR method* OR management* OR traction* OR withdrawal *). #5 accelerat* OR rapid* OR short* OR speed* OR fast OR velocity OR duration OR rate OR time OR "regional accelerated phenomenon" OR RAP. #6 #3 OR #4 OR #5 #7 #1 AND #2 AND #6
PubMed	#1 orthodontic* OR "tooth movement" OR "orthodontic tooth movement" OR "tooth displacement " OR "orthodontic treatment" OR "orthodontic therapy". #2 (canine* OR cuspid) AND (impacted* OR retained* OR transposed* OR ectopia* OR eruption* OR displaced* OR malpositioned* OR unerupted*). #3 (impact*) AND (canine* OR cuspid* (AND (upper* OR maxillary* OR palatally* OR unilateral* OR bilateral*). #4 (impact*) AND (canines* OR cuspids*) AND (maxillary* OR palatally*) AND (treatment* OR exposure* OR technique* OR approach* OR eruption* OR method* OR management* OR traction* OR withdrawal *). #5 accelerat* OR rapid* OR short* OR speed* OR fast OR velocity OR duration OR rate OR time OR "regional accelerated phenomenon" OR RAP. #6 #3 OR #4 OR #5 #7 #1 AND #2 AND #6
Scopus	#1 TITLE-ABS-KEY (orthodontic* OR "tooth movement" OR "orthodontic tooth movement” OR "tooth displacement “OR "orthodontic treatment” OR "orthodontic therapy"). #2 TITLE-ABS-KEY (canine* OR cuspid) AND TITLE-ABS-KEY (impacted* OR retained* OR transposed* OR ectopia* OR eruption* OR displaced* OR malpositioned* OR unerupted*). #3 TITLE-ABS-KEY (impact*) AND TITLE-ABS-KEY (canine* OR cuspid*) AND TITLE-ABS-KEY (upper* OR maxillary* OR palatally* OR unilateral* OR bilateral*). #4 TITLE-ABS-KEY (impact*) AND TITLE-ABS-KEY (canines* OR cuspids*) AND TITLE-ABS-KEY (maxillary* OR palatally*) AND TITLE-ABS-KEY (treatment* OR exposure* OR technique* OR approach* OR eruption* OR method* OR management* OR traction* OR withdrawal *). #5 TITLE-ABS-KEY (accelerat* OR rapid* OR short* OR speed* OR fast OR velocity OR duration OR rate OR time OR "regional accelerated phenomenon" OR RAP). #6 #3 OR #4 OR #5 #7 #1 AND #2 AND #6
Web of Science	#1TS = (orthodontic OR "tooth movement" OR "orthodontic tooth movement” OR "tooth displacement “OR "orthodontic treatment" OR "orthodontic therapy"). #2TS = (canine* OR cuspid) AND TS= (impacted* OR retained* OR transposed* OR ectopia* OR eruption* OR displaced* OR malpositioned* OR unerupted*). #3TS = (impact*) AND TS= (canine* OR cuspid*) AND TS= (upper* OR maxillary* OR palatally* OR unilateral* OR bilateral*). #4TS = (impact*) AND TS = (canines* OR cuspids*) AND TS = (maxillary* OR palatally*) AND TS = (treatment* OR exposure* OR technique* OR approach* OR eruption* OR method* OR management* OR traction* OR withdrawal*). #5TS = (accelerat* OR rapid* OR short* OR speed* OR fast OR velocity OR duration OR rate OR time OR "regional accelerated phenomenon" OR RAP). #6 #3 OR #4 OR #5 #7 #1 AND #2 AND #6
Google Scholar	#1 (orthodontic OR " orthodontic treatment ") AND (impacted OR retained OR transposed OR ectopia OR eruption OR displaced OR malpositioned OR unerupted) AND (canines OR cuspids) AND (upper OR maxillary OR palatal OR palatally OR unilateral OR bilateral). #2 (orthodontic OR " orthodontic treatment ") AND (Impacted OR retained OR transposed OR ectopia OR eruption OR displaced OR malpositioned OR unerupted) AND (canines OR cuspids) AND (upper OR maxillary OR palatal OR palatally OR unilateral OR bilateral) AND (treatment OR exposure OR technique OR approach OR eruption OR method OR management OR traction OR withdrawal). #3 (orthodontic OR " orthodontic treatment ") AND (impacted OR retained OR transposed OR ectopia OR eruption OR displaced OR malpositioned OR unerupted) AND (canines OR cuspids) AND (upper OR maxillary OR palatal OR palatally OR unilateral OR bilateral) AND (treatment OR exposure OR technique OR approach OR eruption OR method OR management OR traction OR withdrawal) AND (acceleration OR accelerating OR accelerated OR rapid OR short OR speed OR fast OR velocity OR duration OR rate OR time OR "regional accelerated phenomenon" OR RAP)
Trip	(orthodontic OR "tooth movement" OR "orthodontic tooth movement" OR "Tooth displacement " OR "orthodontic treatment" OR "orthodontic therapy") AND (Impacted OR retained OR transposed OR ectopia OR eruption OR displaced OR malpositioned OR unerupted) AND (canines OR cuspids) AND (upper OR maxillary OR palatal OR palatally OR unilateral OR bilateral) AND (treatment OR exposure OR technique OR approach OR eruption OR method OR management OR traction OR withdrawal) AND (acceleration OR accelerating OR accelerated OR rapid OR short OR speed OR fast OR velocity OR duration OR rate OR time OR "regional accelerated phenomenon" OR RAP)
OpenGrey	#1 impacted canine AND management. #2 orthodontic AND impacted canine. #3 impacted canines OR impacted cuspids OR unerupted canines OR maxillary impacted canines OR palatally impacted canines OR palatally impacted canines therapy OR palatally impacted canines treatment OR surgical exposure of palatally impacted canines OR Orthodontic traction of impacted maxillary canines OR alignment of impacted permanent maxillary canines OR surgical methods of treating palatally impacted canines OR Closed surgical exposure of palatally impacted canine OR open surgical exposure of palatally impacted canine OR accelerating the orthodontic movement of maxillary canine impaction OR orthodontic treatment acceleration of maxillary canine impaction
PQDT Open (from ProQuest)	#1 (orthodontic OR "management ") AND (impacted OR retained OR transposed OR ectopia OR eruption OR displaced OR malpositioned OR unerupted) AND (canine OR upper canine OR maxillary canine OR palatally canine OR cuspid OR permanent maxillary canines). #2 (orthodontic OR "management") AND (impacted OR retained OR transposed OR ectopia OR eruption OR displaced OR malpositioned OR unerupted) AND (canine OR upper canine OR maxillary canine OR palatally canine OR cuspid OR permanent maxillary canines) AND (treatment OR exposure OR technique OR approach OR eruption OR method OR management OR traction OR withdrawal). #3 (orthodontic OR "management") AND (impacted OR retained OR transposed OR ectopia OR eruption OR displaced OR malpositioned OR unerupted) AND (canine OR upper canine OR maxillary canine OR palatally canine OR cuspid OR permanent maxillary canines) AND (treatment OR exposure OR technique OR approach OR eruption OR method OR management OR traction OR withdrawal) AND (acceleration OR accelerating OR accelerated OR rapid OR short OR speed OR fast OR velocity OR duration OR rate OR time OR "regional accelerated phenomenon" OR RAP)

Study selection and data extraction

The eligibility of the selected trials to be included in this review was evaluated by one reviewer (MRM) and then independently by the second reviewer (MYH), and a third author (OAH) was consulted to resolve conflicts. After the initial evaluation of titles and abstracts, as well as when reading the title or abstract was not sufficient to make the inclusion decision, the full text of relevant articles that appeared to be appropriate for inclusion was evaluated. Articles that did not meet at most one of the inclusion criteria were excluded. Data and study characteristics were extracted from the included trials independently by two authors (MRM and MYH) using predefined extraction tables, and a third author (OAH) was consulted to resolve disagreements. The characteristics tables included the following items: study setting (authors name, publication year, and study setting), study design and type of impaction, materials (sample size, gender, age, and dropouts), intervention (type of appliance, type of attachment, mechanism of traction, and type of withdrawal technique), comparison (type of appliance, type of attachment, mechanism of traction, and type of withdrawal technique), outcome measures, and conclusions.

Assessment of risk of bias in included studies and strength of evidence

The two reviewers (MRM and MYH) evaluated the quality of the included studies using Cochrane’s risk of bias tool (RoB 2 tool) for RCTs and Q-RCTs [37] and by Risk of Bias in Non-randomized Studies of Interventions (ROBINS-I) tool for non-randomized trials [38]. When a lack of consistency was observed, a third author (OAH) was consulted to arrive at a resolution. We evaluated the following fields as at low, high, or unclear risk of bias: sequence generation (selection bias), allocation concealment (selection bias), blinding of participants and personnel (performance bias), blinding of outcome assessors (detection bias), incomplete outcome data addressed (attrition bias), selective outcome reporting (reporting bias), and other sources of bias. Then, the overall risk of bias for each trial included was reported according to the following criteria: (i) low risk of bias: if all fields were evaluated as at low risk of bias (bias improbable to change the results critically). (ii) Unclear risk of bias: if at least one or more fields were assessed as at unclear risk of bias (bias carries some doubt about the results). (iii) High risk of bias: if at least one or more fields were evaluated as at high risk of bias (bias affects the results critically; excluded from the primary analysis). An additional summary of the reliability of the conclusions and strength of evidence was performed using the Grading of Recommendations Assessment, Development, and Evaluation (GRADE) approach [39].

Results

Literature Flow, Study Selection, and Inclusion

**Figure 1:**
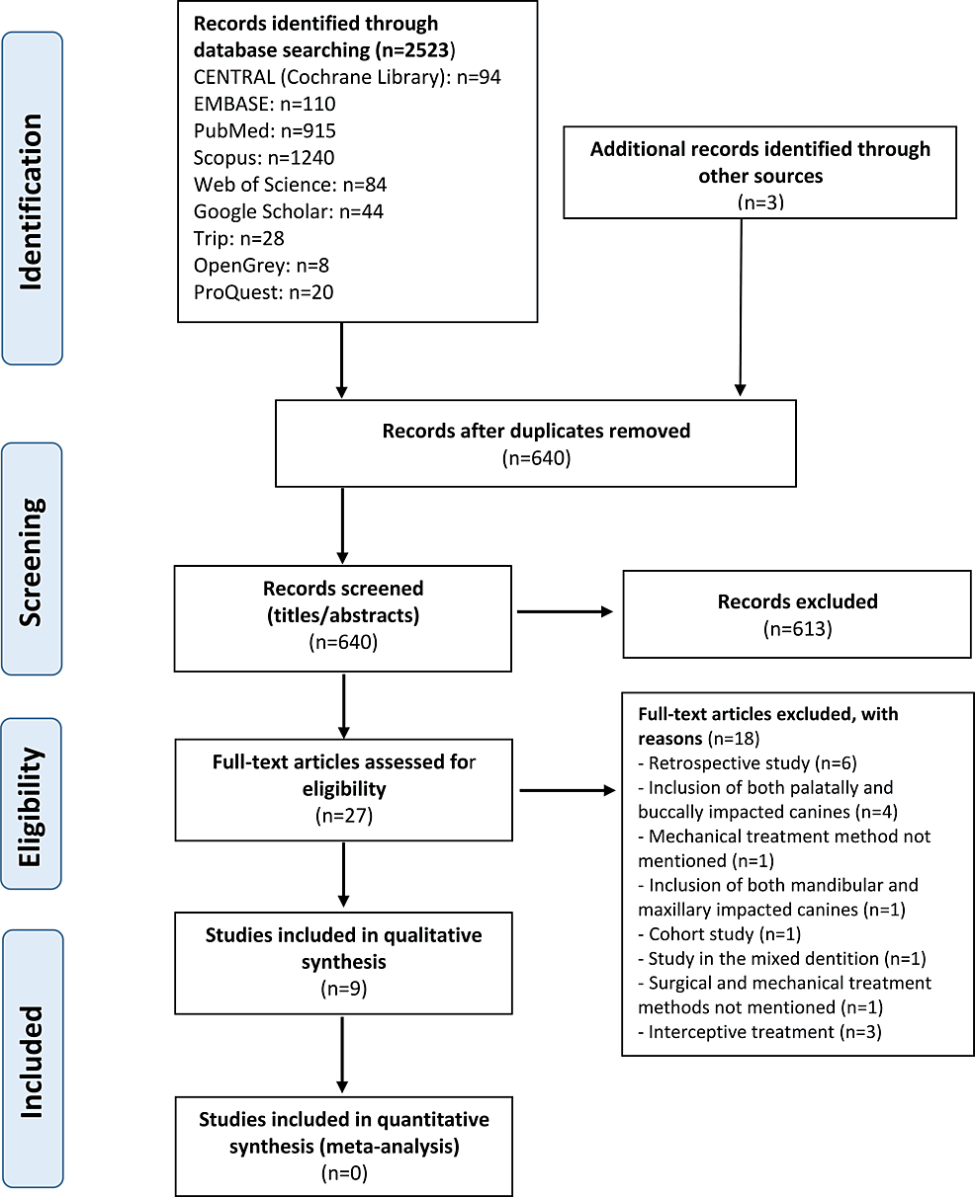
Preferred Reporting Items for Systematic Reviews and Meta-Analyses (PRISMA) flow diagram

The electronic search in the databases revealed 2523 papers, in addition to three papers that were identified by the search performed in reference lists. After taking off the duplicates, 640 articles were reviewed. The titles and abstracts were examined for eligibility, and all papers that failed to meet the eligibility criteria were discarded. As a result, 27 articles were selected to read the full text and 18 of these were excluded. A summary of the excluded articles after full-text assessment with reasons for exclusion is presented in Table 2. Finally, nine studies were included in the systematic review. The PRISMA flow diagram is shown in Figure 1.

**Table 2:** Studies excluded and reasons for exclusion

No.	Study	Reason of excluding
1	Koutzoglou SI, Kostaki A. Effect of surgical exposure technique, age, and grade of impaction on ankylosis of an impacted canine, and the effect of rapid palatal expansion on eruption: A prospective clinical study. Am J Orthod Dentofacial Orthop. 2013 Mar;143(3):342-52. DOI: 10.1016/j.ajodo.2012.10.017. PMID: 23452968.	The study included mandibular and maxillary impacted canines
2	Crescini A, Nieri M, Buti J, Baccetti T, Mauro S, Prato GP. Short- and long-term periodontal evaluation of impacted canines treated with a closed surgical-orthodontic approach. J Clin Periodontol. 2007 Mar;34(3):232-42. DOI: 10.1111/j.1600-051X.2006.01042.x. Epub 2007 Jan 25. PMID: 17257160.	Cohort study
3	Kocsis A, Seres L. Orthodontic screws to extrude impacted maxillary canines. J Orofac Orthop. 2012 Jan;73(1):19-27. DOI: 10.1007/s00056-011-0057-9. Epub 2012 Jan 12. PMID: 22234413.	The study included palatally and buccally impacted canines
4	Zasciurinskiene E, Bjerklin K, Smailiene D, Sidlauskas A, Puisys A. Initial vertical and horizontal position of palatally impacted maxillary canine and effect on periodontal status following surgical-orthodontic treatment. Angle Orthod. 2008 Mar;78(2):275-80. DOI: 10.2319/010907-8.1. PMID: 18251594.	Retrospective study
5	Crescini A, Nieri M, Buti J, Baccetti T, Pini Prato GP. Orthodontic and periodontal outcomes of treated impacted maxillary canines. Angle Orthod. 2007 Jul;77(4):571-7. DOI: 10.2319/080406-318.1. PMID: 17605500.	The study included palatally and buccally impacted canines
6	Ferguson DJ, Rossais DA, Wilcko MT, Makki L, Stapelberg R. Forced-eruption time for palatally impacted canines treated with and without ostectomy-decortication technique. Angle Orthod. 2019 Sep;89(5):697-704. DOI: 10.2319/111418-809.1. Epub 2019 Mar 19. PMID: 30888841; PMCID: PMC8111832.	Retrospective study
7	Sigler LM, Baccetti T, McNamara JA Jr. Effect of rapid maxillary expansion and transpalatal arch treatment associated with deciduous canine extraction on the eruption of palatally displaced canines: A 2-center prospective study. Am J Orthod Dentofacial Orthop. 2011 Mar;139(3):e235-44. DOI: 10.1016/j.ajodo.2009.07.015. PMID: 21392667.	Study in the mixed dentition
8	Becker A, Chaushu S. Success rate and duration of orthodontic treatment for adult patients with palatally impacted maxillary canines. Am J Orthod Dentofacial Orthop. 2003 Nov;124(5):509-14. DOI: 10.1016/s0889-5406(03)00578-x. PMID: 14614417.	Retrospective study
9	Schubert M, Baumert U. Alignment of impacted maxillary canines: a critical analysis of eruption path and treatment time. J Orofac Orthop. 2009 May;70(3):200-12. English, German. DOI: 10.1007/s00056-009-0901-3. Epub 2009 May 31. PMID: 19484413.	Retrospective study
10	Oz AZ, Ciger S. Health of periodontal tissues and resorption status after orthodontic treatment of impacted maxillary canines. Niger J Clin Pract. 2018 Mar;21(3):301-305. DOI: 10.4103/njcp.njcp_419_16. PMID: 29519977.	Clinical study, but treatment methods (surgical and mechanical) were not mentioned
11	Schmidt AD, Kokich VG. Periodontal response to early uncovering, autonomous eruption, and orthodontic alignment of palatally impacted maxillary canines. Am J Orthod Dentofacial Orthop. 2007 Apr;131(4):449-55. DOI: 10.1016/j.ajodo.2006.04.028. PMID: 17418710.	Clinical study, but mechanical treatment method was not mentioned
12	Mummolo S, Nota A, De Felice ME, Marcattili D, Tecco S, Marzo G. Periodontal status of buccally and palatally impacted maxillary canines after surgical-orthodontic treatment with open technique. J Oral Sci. 2018 Dec 27;60(4):552-556. DOI: 10.2334/josnusd.17-0394. Epub 2018 Jul 9. PMID: 29984786.	The study included palatally and buccally impacted canines
13	Baccetti T, Leonardi M, Armi P. A randomized clinical study of two interceptive approaches to palatally displaced canines. Eur J Orthod. 2008 Aug;30(4):381-5. DOI: 10.1093/ejo/cjn023. Epub 2008 Jun 3. PMID: 18524761.	Interceptive treatment
14	Leonardi M, Armi P, Franchi L, Baccetti T. Two interceptive approaches to palatally displaced canines: a prospective longitudinal study. Angle Orthod. 2004 Oct;74(5):581-6. DOI: 10.1043/0003-3219(2004)074<0581:TIATPD>2.0.CO;2. PMID: 15529490.	Interceptive treatment
15	Baccetti T, Sigler LM, McNamara JA Jr. An RCT on the treatment of palatally displaced canines with RME and/or a transpalatal arch. Eur J Orthod. 2011 Dec;33(6):601-7. DOI: 10.1093/ejo/cjq139. Epub 2010 Nov 8. PMID: 21059877.	Interceptive treatment
16	Caminiti MF, Sandor GK, Giambattistini C, Tompson B. Outcomes of the surgical exposure, bonding and eruption of 82 impacted maxillary canines. J Can Dent Assoc. 1998 Sep;64(8):572-4, 576-9. PMID: 9785687.	The study included palatally and buccally impacted canines
17	Caprioglio A, Vanni A, Bolamperti L. Long-term periodontal response to orthodontic treatment of palatally impacted maxillary canines. Eur J Orthod. 2013 Jun;35(3):323-8. DOI: 10.1093/ejo/cjs020. Epub 2012 Apr 24. PMID: 22531665.	Retrospective study
18	Pearson MH, Robinson SN, Reed R, Birnie DJ, Zaki GA. Management of palatally impacted canines: the findings of a collaborative study. Eur J Orthod. 1997 Oct;19(5):511-5. DOI: 10.1093/ejo/19.5.511. PMID: 9386337.	Retrospective study

Characteristics of the Included Studies

The characteristics of the included trials are given in Tables 3, 4. Five completed RCTs, three Q-RCTs, and one CCT, including 371 patients and 385 PICs, were included in this systematic review. The age of patients ranged from 11.1 to 34 years. All studies were of a parallel group design except for one study, which was of a split-mouth design [31]. Seven studies compared one traction technique with another technique without any attempt to accelerate the treatment [7,12,15,17,40-42], whereas acceleration of orthodontic traction was the experimental group in two RCTs [31,43]. These studies were published between 2007 and 2018. The countries of origin of the included studies were the United States of America, Italy, Jordan, Iran, Sweden, Egypt, and the United Kingdom. All of these studies were in English.

**Table 3:** Characteristics of the included studies (conventional non-accelerated traction studies) RCT: randomized clinical trial; Q-RCT: quasi-randomized trial; M: males; F: females; PDC: palatally displaced canine; TPA: transpalatal arch; BOP: bleeding on probing; CEJ: cementoenamel junction; TADs: temporary anchorage devices.

Study/setting	Study design	Intervention (type of appliance, type of attachment, mechanism of traction, type of withdrawal technique)	Comparison (type of appliance, type of attachment, mechanism of traction, type of withdrawal technique)	Outcome measures	Conclusions
Smailiene et al. (2013) [7], Sweden	Q-RCT, unilateral PDC	Fixed appliances, open surgical approach, and free eruption	Fixed appliances, ballista loop, and closed flap surgery	Duration of orthodontic traction, duration of orthodontic treatment, probing pocket depth, the width of keratinized tissue, gingival recession, bone support	There were no significant differences in the post-treatment periodontal status of the canines and adjacent teeth either by open surgery with free eruption or by closed flap technique
Parkin et al. (2012) [17], UK	RCT, unilateral PDC	Fixed appliances, eyelet attachment with a golden chain, and open surgical exposure	Fixed appliances, eyelet attachment with a golden chain, and closed surgical exposure	Actual surgical time, patient-reported outcome	There was no difference in the operating time between the open and closed surgical techniques. There were no differences in any of the patient-reported outcomes between the two surgical procedures. Most participants reported pain, discomfort, impairment to everyday activities, and the need for regular analgesia after surgical exposure were of short duration and subsided after a few days
Heravi et al. (2016) [15], Iran	CCT, unilateral or bilateral PDC	Fixed appliances, two miniscrews, cantilever springs, and open surgical exposure	Fixed appliances, trans palatal arch (TPA), cantilever spring, and open surgical exposure	Canine and lateral incisor root resorption, BOP, gingival index, patient’s pain experience, duration of orthodontic traction	The mean duration of the forced eruption was 5.2 months in the control group and 5.1 months in the experimental group. The clinical success rate was 100%. TADs allow a more controlled movement of the impacted tooth
Gharaibeh and Al-Nimri(2008) [12], Jordan	Q-RCT, unilateral PDC	Fixed appliances, a golden chain, and closed surgical exposure	Fixed appliances, lingual button, a golden chain, and open surgical exposure	Duration of surgery and the patient’s perception of pain	The mean surgical duration for the open-eruption technique was 30.9 ± 10.1 minutes compared with 37.7 ± 8.4 minutes for the closed-eruption technique. This difference was statistically significant (p = 0.006). On the first postoperative day, six patients (33%) in the close-eruption group reported severe pain compared with four patients (22%) in the open-eruption group. This difference was not statistically significant (p = 0.123)
Björksved et al. (2018) [40], Sweden	RCT, unilateral or bilateral PDC	Fixed appliances, attachment with a chain, and closed surgical technique	Fixed appliances and open surgical technique	Surgery time, complications, and patients’ perceptions	No statistically significant differences in surgery time between the two groups. Postoperative complications were similar between the groups in unilateral PDCs but more common in the open group in bilateral cases. More patients in the open group experienced pain and impairment compared to the closed group
Parkin et al.(2013) [41], UK	RCT, unilateral PDC	Fixed appliances, twin-wire technique or elastic chain, and open surgical exposure	Fixed appliances, twin-wire technique or elastic chain, and closed surgical exposure	Clinical periodontal attachment level, crown height, gingival recession, radiographic alveolar bone levels, duration of orthodontic traction	Duration: open exposure - 10.2 months (SD: 4.2); closed exposure - 13.2 months (SD: 8.5). Exposure and alignment of the PDCs have a small impact on periodontal health
Smailiene et al. (2013) [42], Sweden	Q-RCT, unilateral PDC	Fixed appliances, open surgical approach, and free eruption	Fixed appliances, ballista loop, and closed flap surgery	Post-treatment status (radiological, periodontal, and intraoral examination), visual assessment of the color and shape of the crown, inclination, position in the dental arch, and function) of palatally impacted canines	The post-treatment status of the palatally impacted and the adjacent teeth after the surgical-orthodontic treatment did not significantly differ between the groups treated with the two different surgical methods (open and closed). Both treatment methods can be considered acceptable for the treatment of palatally impacted canines

**Table 4:** Characteristics of the included studies that evaluated accelerated traction RCT: randomized clinical trial; M: males; F: females; PDC: palatally displaced canine.

Study/setting	Study design	Sample size, gender, age (years)	Intervention (type of appliance, type of the attachment, mechanism of traction, type of withdrawal technique)	Comparison (type of appliance, attachment, traction mechanism, withdrawal technique)	Outcome measures	Main findings
Fischer (2007) [31], USA	RCT, bilateral PDC	6 (2M + 4F), 12 impacted canines. 11.1-12.9 years	Fixed appliances, corticotomy-assisted, and open surgical technique	Fixed appliances, conventional withdrawal, and open surgical technique	Treatment time, velocity	The reduction in treatment time ranged from 28% to 33%. The corticotomy-assisted canines moved at a rate of 1.06 mm/mo vs. 0.75 mm/mo for the conventional canines
Dehis et al. (2018) [43], Egypt	RCT, unilateral PDC	12 (3M + 9F) 16-34 years	Fixed appliances, intra-epidermic vitamin C injection, power chain, and closed surgical technique	Fixed appliances, power chain, and closed surgical technique	Rate of the orthodontic tooth movement, the width of keratinized tissues, alveolar bone thickness, lateral incisor root resorption	The rate of tooth movement was recorded in the intervention group (2-2.5 mm), compared to the control group (0.5-1.5 mm)

Two studies were composed of the same sample [7,42] evaluating different outcomes. The sample size ranged from 12 [31] to 119 [40] PICs. One study included only bilateral PICs [31], six studies included only unilateral PICs [7,12,17,40,41,43], and the last two studies included both types (unilateral and bilateral) of impaction [15,40]. Out of the nine included studies, six studies compared the closed with the open-eruption method, two studies used the open-eruption method in the two compared groups [15,31], and one study used the closed-eruption method in the two compared groups [43].

The distribution of men and women was described in all studies (106 males and 265 females). One study included only female patients [15], whereas the number of females was more than that of males in the other eight studies.

Two of the included studies compared an acceleration method with a conventional method of PIC treatment [31,43], three studies compared a conventional method with the free eruption of impacted canines [7,40,41], while the other four studies compared a conventional method with another conventional method of PIC traction.

The included studies used several mechanical orthodontic traction methods for the withdrawal of the impacted canines. Two studies used the power chain [41,43], two studies used the golden chain [16,17], two studies used the ballista loop [7,42], one study used the twin-wire technique [41], one study used the cantilever spring [15], and the remaining two studies did not mention the used mechanism for traction [31,40].

Risk of Bias of the Included Studies

**Figure 2:**
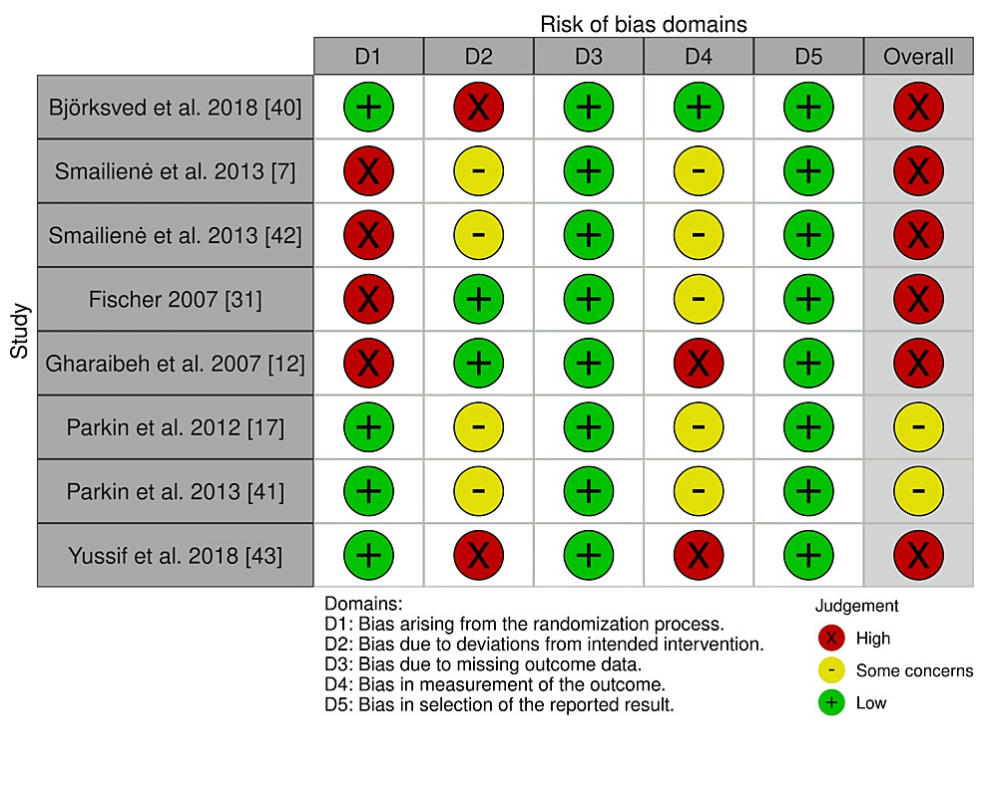
Risk of bias summary: review authors' judgments about each risk of bias item for each included study

**Figure 3:**
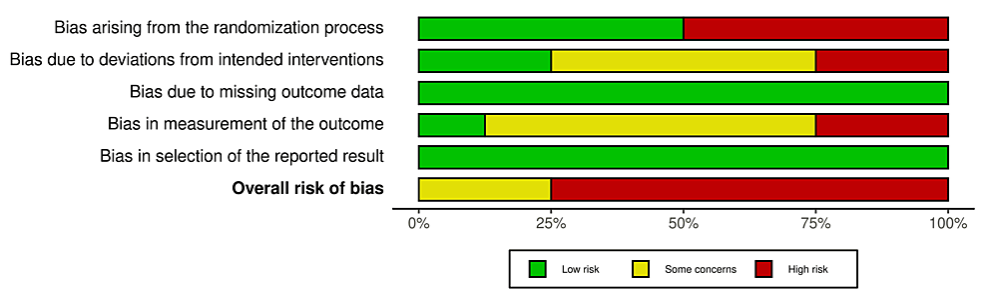
The overall risk of bias for each domain

A summary of the risk of bias in the included studies is presented in Figures 2, 3. Six studies out of the included RCTs were at high risk of bias [7,12,31,40,42,43], and the other two studies were at a "some concern" of bias [17,41]. Participants’ blinding and randomization process was the most problematic field. The risk of bias assessment and the overall risk of bias for each domain of the CCT study [15] are presented in Table 5. The study was at a high risk of bias. More details about the risk of bias assessment with supporting reasons beyond each assessment of the included RCTs are given in Table 6.

**Table 5:** Risk of bias of the included controlled clinical trial in the systematic review

Study	Bias due to confounding	Bias in the selection of participants for the study	Bias in the classification of interventions	Bias due to deviations from intended interventions	Bias due to missing data	Bias in the measurement of outcomes	Bias in the selection of the reported result	Overall
Heravi et al. (2016) [15]	Low. No confounding is expected	Low. All participants who would have been eligible for the target trial were included in the study, and for each participant, the start of follow-up and start of intervention coincided	No information	Low. There were no deviations from the intended interventions (in terms of implementation or adherence) that were likely to impact the outcome	Low. No dropouts were reported	Serious. The outcome measure was probably influenced by knowledge of the intervention received by study participants	Low. The protocol was not registered. But the pre-defined outcomes mentioned in the methods section seemed to have been reported	Serious

**Table 6:** Risk of bias of included randomized controlled trials in the systematic review

Study	Randomization process	Deviations from intended interventions	Missing outcome data	Measurement of the outcome	Selection of the reported result	Overall bias
Bjorksved et al. (2018) [40]	Low risk. Study participants were randomly allocated in blocks of different sizes, according to a computer-generated randomization list, using concealed allocation	High-risk. Blinding of participants and people delivering the intervention cannot be performed. We judge that the outcome is influenced by a lack of blinding	Low risk. No dropouts were reported	Low risk. “Single blinding was employed in this trial regarding outcome measure assessment and data analysis”	Low risk. The protocol for the study was registered on ClinicalTrials.gov (study ID: NCT02186548) and the outcomes mentioned in the protocol have been reported	High risk
Smailiene et al. (2013) [7]	High risk. No random element was used in generating the allocation sequence. "Every second patient was assigned to one of the study groups"	Some concerns. Blinding of participants and people delivering the intervention cannot be performed. We judge that the outcome might be influenced by a lack of blinding	Low risk. No dropouts were reported	Some concerns. No details of blinding the measurement stage. "The post-treatment examination was performed by other authors"	Low risk. The protocol was not registered. But the pre-defined outcomes mentioned in the methods section seemed to have been reported	High risk
Smailiene et al. (2013) [42]	High risk. No random element was used in generating the allocation sequence. "Every second patient was assigned to one of the study groups"	Some concerns. Blinding of participants and people delivering the intervention cannot be performed. We judge that the outcome might be influenced by a lack of blinding	Low risk. No dropouts were reported	Some concerns. No details of blinding the measurement stage. "The post-treatment examination was performed by other authors"	Low risk. The protocol was not registered. But the pre-defined outcomes mentioned in the methods section seemed to have been reported	High risk
Fischer (2007) [31]	High risk. No mention of the method used for randomization	Low risk. Blinding of participants and people delivering the intervention cannot be performed. We judge that the outcome is not likely to be influenced by a lack of blinding	Low risk. No dropouts were reported	Some concerns. No details of blinding the measurement stage. "The orthodontist had no knowledge as to which canine had the corticotomy procedure"	Low risk. The protocol was not registered. But the pre-defined outcomes mentioned in the methods section seemed to have been reported	High risk
Gharaibeh and Al-Nimri (2008) [12]	High risk. No mention of the method used for randomization. "The choice of the exposure type was randomly selected"	Low risk. Blinding of the participating patient and the treating clinician was not possible due to the nature of the trial	Low risk. No dropouts were reported	High risk. No details of blinding the measurement stage (data collection) and data analyzer	Low risk. The protocol was not registered. But the pre-defined outcomes mentioned in the methods section seemed to have been reported	High risk
Parkin et al. (2012) [17]	Low risk. The randomization was undertaken using computer-generated random numbers to ensure that equal numbers were allocated to each intervention and allocation concealment was with consecutively-numbered, sealed, opaque envelopes	Some concerns. No details about blinding, either the patient or clinician of the type of mechanical intervention used	Low risk. No dropouts were reported	Some concerns. The examiners were masked as to the patient’s group allocation when undertaking the clinical examinations. The patient details were removed from all study models and radiographs, which were only labeled with the participant randomization number. No details about blinding the examiners or the type of mechanical intervention used	Low risk. The protocol was not registered. But the pre-defined outcomes mentioned in the methods section seemed to have been reported	Some concerns
Parkin et al. (2013) [41]	Low risk. The randomization was undertaken using computer-generated random numbers in randomly allocated blocks of 2, 4, 6, and 8 to ensure that there were equal numbers allocated to each intervention. Allocation concealment was with consecutively numbered, sealed, opaque envelopes	Some concerns. Blinding of participants and people delivering the intervention cannot be performed. We judge that the outcome might be influenced by a lack of blinding	Low risk. No dropouts were reported	Some concerns. No details of blinding the measurement stage (data collection) and data analyzer. "A masked assessor probably be able to guess which canine was previously impacted, owing to positional differences, but would not be able to tell which technique was used"	Low risk. The protocol was not registered. But the pre-defined outcomes mentioned in the methods section seemed to have been reported	Some concerns
Dehis et al. (2018) [43]	Low risk. Randomization was carried out by using computer-generated random numbers	High risk. No blinding, but we judge that the outcome is influenced by the lack of blinding	Low risk. No dropouts were reported	High risk. No details of blinding the measurement stage (data collection) and data analyzer	Low risk. The protocol for the study was registered on ClinicalTrials.gov (study ID: NCT03260829) and the outcomes mentioned in the protocol have been reported	High risk

Effects of interventions

The main findings of the included studies are given in Table 7. The results will be reported under the following two main themes: (1) comparison between different techniques without acceleration and (2) comparison between a conventional technique versus an accelerated technique.

**Table 7:** The main findings of the studies included in this systematic review OST: open surgical technique; CST: closed surgical technique; PPD: periodontal pocket depth; GR: gingival recession; KT: width of the keratinized tissue; BS: bone support; CAL: clinical attachment level; GI: gingival index.

Study	Groups	Velocity and duration of orthodontic traction/treatment	Periodontal outcomes/root resorption	Patient-reported outcomes
Smailiene et al. (2013) [7]	OST vs. CST group	Mean treatment time was 28.41 ± 4.96 and 32.19 ± 11.73 months in OST and CST group, respectively (p > 0.05). Mean eruption/extrusion time was 3.05 ± 1.07 and 6.86 ± 4.53 months for OST and CST group, respectively (p < 0.01)	Mean PPD was 2.2 ± 0.55 mm and 2.01 ± 0.42 mm on the impacted canine side and contralateral side, respectively (p < 0.05), with no significant differences in PPD and BS between test groups. No significant differences in GR and KT between groups and between the test and contralateral sides. In comparison with the contralateral side, differences were found in BS at the mesial side of the canine and the distal side of the lateral incisor	Not evaluated
Smailiene et al. (2013) [42]	OST vs. CST group	Not evaluated	No significant differences in PPD between test groups. Mean PPD on the impacted canine side was 2.14 mm (SD = 0.38) and 2.28 mm (SD = 0.69) in the OST and CST group, respectively, while, on the contralateral side, it was 1.95 mm (SD = 0.38) and 2.20 mm (SD = 0.42) in the OST and CST group, respectively. No significant differences in GR between groups and between the test and contralateral sides. BS did not differ significantly between the groups (mean bone support of 89.33%; SD = 6.87%) in the OST group and (86.66%; SD = 6.94%) in the CST group	Not evaluated
Parkin et al. (2012) [17]	OST vs. CST group	Not evaluated	Not evaluated	In the two groups, the pain lasted for “several days” in 60% of the sample. Three patients in the CST group and six patients in the OST group reported that the pain lasted for more than several days, but this was not statistically significant. Twenty-eight of 31 participants (90%) in the OST group required pain relief compared with 23 of 29 (79%) in the CST group, which was not statistically significant. The difference in pain duration between groups was not significant (p = 0.161)
Parkin et al. (2013) [41]	OST vs. CST group	Duration of active traction: 10.2 months; SD = 4.2 and 13.2 months; SD = 8.5 in OST and CST group, respectively	Mean CAL difference between OST and CST groups was 0.1 mm (open: 0.5 mm, SD = 0.8; closed: 0.6 mm, SD = 0.6; p = 0.782). Eight subjects (28%) in the CST group and 12 subjects (36%) in the OST group showed root visibility at the mid-palatal point between zero and 2 mm (P = 0.464). In the CST group, nine subjects (31%) showed recession of at least 1 mm on the mid-buccal aspect of the operated canine (1 mm in seven subjects, 2 mm in subjects). In the OST group, eight subjects (24%) showed recession of at least 1 mm (1 mm in five and 2 mm in three subjects; p = 0.774)	Not evaluated
Heravi et al. (2016) [15]	Two miniscrews vs. transpalatal arch	The mean eruption time was 5.2 months in the control group and 5.1 months in the experimental group (p = 0.125)	No significant difference in the volume of canine root resorption and in GI between the two groups (p = 0.937). The volume of lateral incisor root resorption in the control group was significantly greater than in the experimental group (nearly fourfold)	After three weeks, higher pain levels were reported in the control group (p = 0.012); but, at the end of treatment, this difference was not statistically significant (p = 0.769). In the experimental group, the pain level was determined one day after the placement of miniscrews, and the mean value was 2.1
Gharaibeh and Al-Nimri(2008) [12]	OST vs. CST group	Not evaluated	Not evaluated	On the first postoperative day, six patients (33%) in the CST group and four patients (22%) in the OST group reported severe pain (p = 0.123). On the second postoperative day, only two patients in the OST group continued to experience severe pain whereas none in the CST group reported severe pain. Neither group reported any severe pain after the second postoperative day
Björksved et al. (2018) [40]	OST vs. CST group	Not evaluated	Not evaluated	The number of surgical complications within four weeks post-surgery was similar in the two groups. On the evening of operation day, significantly higher pain scores were at the injection of local anesthetics in the CST group, while post-surgery pain showed significantly higher pain scores in the OST group. Significantly more pain level (p = 0.010) in the seven days post-surgery was in the OST group
Fischer (2007) [31]	Corticotomy-assisted canine treatment vs. conventional treatment	Significantly higher tooth movement velocity was recorded in all corticotomy-assisted canines and the treatment time was reduced by 28-33%	No clinical differences were recorded in the periodontal probing and bone levels between the two groups	Not evaluated
Dehis et al. (2018) [43]	Vitamin C injection (intervention group) vs. conventional traction (control group)	Statistically, a greater mean area percent of the movement rate was recorded in the intervention group compared to the control group (1.08 ± 0.376, 2.25 ± 0.274, respectively; p < 0.003). Clinically, significant improvement was reported in the movement rate in the intervention group (2-2.5 mm/month) compared with the control group (0.5-1.5 mm/month)	No intra- (p = 1.000) or intergroup statistical significant difference (p = 0.416) was reported in the KT between the pre- and postoperative values. No statistically significant differences were found in the gingival margin level between both groups. The intragroup analysis showed statistically significant differences in the alveolar bone thickness (p = 0.000). While the intergroup analysis of the postoperative results in both groups showed statistically and radiographically significant differences (p = 0.002)	Not evaluated

The high clinical heterogeneity among the retrieved studies (variability in the applied interventions, in the studies' designs, in the studied outcomes, and in the patient's ages) did not allow for conducting a quantitative synthesis of the data in a meta-analysis.

Theme 1: Conventional Technique Versus Another Technique Without Acceleration

Duration of orthodontic traction: Three articles reported the duration of orthodontic traction of PICs as our primary outcome for this systematic review [7,15,41]. Smailiene et al. [7] used the closed surgical technique with the ballista loop (as a mechanical method of traction) versus the open surgical technique with the free eruption in a parallel group-design RCT. This study was performed on 43 patients treated for unilateral PICs, with a mean age of 18.6 ± 3.45 years in the open eruption group and 19.7 ± 4.37 years in the closed eruption group. They reported that the mean duration of canine traction was greater in the closed surgery group than in the open technique group (6.86 ± 4.53 and 3.05 ± 1.07 months, respectively, p < 0.01). They assessed the duration from the surgical exposure until the bonding of a bracket in the middle of the canine's labial surface.

On the other hand, Parkin et al. [41] investigated the differences in the periodontal outcomes of unilateral palatally displaced canines (PDCs) exposed with either an open or closed surgical technique in a parallel group design RCT. They used the twin-wire or elastic chain technique for impacted canine traction. This study was performed on 62 participants, with a mean age of 14.2 years for the open surgical exposure group and 14 years for the closed surgical exposure group. They found that the duration of active traction of impacted canines was 10.2 months with open exposure and 13.2 months with closed exposure.

In addition, Heravi et al. [15], in a parallel group design CCT, evaluated the movement of unilateral PICs away from the roots of neighboring teeth, to decrease undesired side effects on adjacent teeth. This study was performed on 34 patients with a mean age of 15.6 ± 2.1 years old, using two miniscrews versus transpalatal arch (TPA). They used the cantilever springs as a mechanical method of traction and the open surgical exposure technique in both study groups. They reported that the mean duration of the forced eruption was 5.2 months in the control group (TPA group) and 5.1 months in the experimental group (miniscrews group) with no statistically significant difference between these two groups.

Duration of complete orthodontic treatment: One article reported the duration of complete orthodontic treatment [7]. The prospective study of Smailiene et al. concluded that the duration of treatment was greater in the closed surgery group than in the open technique group, but the difference between these two groups was not significant (28.41 ± 4.96 months for the open technique group and 32.19 ± 11.73 months for the closed surgery group, p > 0.05).

Periodontal outcomes: Four articles evaluated the periodontal status of the withdrawn impacted canines [7,15,41,42]. The two articles of Smailiene et al. [7,42] concluded that there was no statistical difference in terms of mean pocket depth, gingival recession, bone support, and width of keratinized gingiva between the open and closed technique groups. Parkin et al. [41] investigated the level of attachment, crown height, bone support, and gingival recession between previously impacted canines and their normal contralateral ones for closed- and open-eruption techniques. The results of this study indicated that there were no statistical differences between the two eruption techniques in the variables assessed. Similarly, in the study of Heravi et al. [15], gingival index (GI) and bleeding on probing (BOP) of impacted canines were determined after the forced eruption and compared between the two groups. The results of this study showed no significant difference between the two groups.

Patient-reported outcomes: Perception of pain after surgical exposure to canines was investigated in four articles [12,15,17,40]. Gharaibeh and Al-Nimri [12] compared the patient’s perception of pain after closed and open surgical exposure of unilateral PICs, using a gold chain technique for impacted canine's traction, in a parallel group design RCT. They assessed the worst pain in their sample of 32 patients (with a mean age of 17.3 for the open surgical exposure group and 17.6 years for the closed surgical exposure group) for seven days after surgery using a numerical rating scale. They found no differences in the perceptions of pain in individuals treated with an open or closed technique. Heravi et al. [15] reported that the mean values of patient-perceived pain, measured by a visual analog scale (VAS), were not different between the two study groups.

Similarly, Parkin et al. [17] investigated the differences in surgical outcomes between open and closed exposure for unilateral PDCs in a parallel group design RCT using a gold chain traction method for impacted canines. This study was performed on 71 participants, with a mean age of 14.3 years for open surgical exposure and 14.1 years for the closed exposure group. The patient-reported outcome consisted of a postoperative questionnaire, which was given to participants at their 10-day surgical review appointment. They reported that there was no difference in the amount of pain between the two study groups (p > 0.05).

Björksved et al. [40] compared the complications and patients’ perceptions between closed and open surgical techniques of unilateral or bilateral PICs in a parallel group design RCT (119 patients, with a mean age of 13.4 years). Patient perception of pain was analyzed from two questionnaires, one in the evening on the day of operation and one seven days post-surgery. The main findings of this study were that patients experienced significantly more post-surgery pain and impairment in the open surgical technique group than in the closed surgical technique group (p = 0.010).

Root resorption: One study reported on root resorption. Heravi et al. [15] found no statistically significant difference in the volume of canine root resorption between the control and experimental groups (x ®= 1.8113 mm3 and x ®= 2.0589 mm3, respectively, p = 0.561). While the volume of lateral incisor root resorption in the control group was significantly greater (nearly fourfold) than in the experimental group (x ®= 5.9060 mm3 and x ®= 1.5211 mm3, respectively, p < 0.001).

Theme 2: Conventional Method of Traction Versus an Accelerated Technique

Duration of complete orthodontic treatment: Only one study reported the total treatment time of six patients with bilateral palatally impacted maxillary canines [31]. Fischer evaluated the effect of "corticotomy-assisted" versus "conventional surgical technique" in a split-mouth RCT and found a reduction of treatment time of about 28-33% [31]. The mean treatment time in this study was 46 weeks for corticotomy-treated canines and 66.7 weeks for conventionally treated canines. The corticotomy procedure was performed only once directly when applying the surgical exposure of one canine.

The velocity of impacted canine movement: Regarding the rate of impacted canine movement, the studies of Fischer and Dehis et al. reported a greater canine movement rate in the acceleration groups [31,43]. Fischer [31] reported that the corticotomy-assisted canines moved at a rate of 1.06 mm/month vs. 0.75 mm/month for the conventional canine's traction. Similarly, Dehis et al. estimated the efficiency of the locally injected vitamin C in the enhancement of the PIC movement in a parallel group design RCT, using the power chain as a traction method for impacted canines [43]. This study was performed on 12 participants aged between 16 and 34 years old. Patients were followed up for one year from the beginning of traction and the injection visits were repeated every two weeks (two visits per month). They reported that there was a clinically significant difference in the rate of impacted canine movement between the intervention group and the control group (2-2.5 mm/month and 0.5-1.5 mm/month, respectively, p < 0.003).

Periodontal outcomes: Two studies evaluated the periodontal outcomes following canine traction and alignment [31,43]. Fischer [31] reported no clinical differences between the corticotomy-assisted canines and their contralateral teeth regarding the periodontal probing and bone levels. Dehis et al. [43] reported that there was no statistical difference between the two study groups in terms of the width of keratinized gingiva and gingival recession variables, while a statistical difference was found in the alveolar bone thickness between the two groups (p = 0.002).

Strength of the Evidence in the Collected Data

Based on the GRADE recommendations, the strength of evidence for the duration of orthodontic traction, periodontal outcomes, and patient’s perception of pain ranged from low to very low, while it was very low for the velocity of impacted canine movement and root resorption, as shown in Tables 8, 9. The decline in the strength of the evidence occurred because of the imprecision, and unclear or high risk of bias.

**Table 8:** Summary of findings according to the GRADE guidelines for the included studies * The risk in the intervention group (and its 95% CI) is based on the assumed risk in the comparison group and the relative effect of the intervention (and its 95% CI). ^a^ Decline two levels for risk of bias in [7,42] (unclear risk of bias of randomization process, unclear risk of bias of deviation from intended intervention, unclear risk of bias in the measurement of outcomes ), and one level for imprecision*. ^b^ Decline one level for risk of bias in [17,41] (unclear risk of bias of deviation from intended intervention), and one level for imprecision**. ^c^ Decline two levels for risk of bias in [15] (unclear risk of bias in classification of interventions, unclear risk of bias in the measurement of outcomes), and one level for imprecision**. ^d^ Decline two levels for risk of bias in [12] (unclear risk of bias of randomization process, unclear risk of bias in the measurement of outcomes), and one level for imprecision**. ^e^ Decline one level for risk of bias in [40] (unclear risk of bias of deviation from intended intervention) and one level for imprecision**. ** Limited number of trials and limited sample size. GRADE: Grading of Recommendations Assessment, Development, and Evaluation.

Patient or population: patients with palatally impacted canines; Intervention: conventional technique of canine retraction; Comparison: another technique of canine retraction without acceleration			
Outcomes	No. of participants (studies)	Certainty of the evidence (GRADE)	Comments
Duration of orthodontic traction	43 (1 RCT)	⨁⊝⊝⊝ Very low^ a^	The evidence is very uncertain about the effectiveness of the ballista loop traction method on the duration of traction
Duration of orthodontic traction	62 (1 RCT)	⨁⨁⊝⊝ Low ^b^	The evidence is uncertain about the effectiveness of the twin-wire technique or elastic chain traction method on the duration of traction
Duration of orthodontic traction	15 cases, 11 controls (1 observational study)	⨁⊝⊝⊝ Very low^ c^	The evidence is very uncertain about the effectiveness of the cantilever spring traction method on the duration of traction
Duration of complete orthodontic treatment	43 (1 RCT)	⨁⊝⊝⊝ Very low ^a^	The evidence is very uncertain about the effectiveness of the ballista loop traction method on the duration of complete orthodontic treatment
Periodontal outcomes	43 (1 RCT)	⨁⊝⊝⊝ Very low^ a^	The evidence suggests no statistical difference in terms of mean pocket depth, gingival recession, bone support, and width of keratinized gingiva between the two surgical and mechanical techniques
Periodontal outcomes	62 (1 RCT)	⨁⨁⊝⊝ Low ^b^	The evidence suggests that no statistical differences in the variables were assessed between the two eruption techniques using the twin-wire technique or elastic chain
Periodontal outcomes	15 cases, 11 controls (1 observational study)	⨁⊝⊝⊝ Very low^ c^	The evidence suggests no significant difference in the variables assessed between the two eruption techniques using cantilever springs
Patient’s perception of pain	32 (1 RCT)	⨁⊝⊝⊝ Very low^ d^	The evidence suggests no differences in the perceptions of pain in individuals treated with an open or closed technique using a golden chain
Patient’s perception of pain	119 (1 RCT)	⨁⨁⊝⊝ Low^ e^	The evidence suggests more post-surgery pain and impairment in the open surgical technique than in the closed surgical technique
Patient’s perception of pain	71 (1 RCT)	⨁⨁⊝⊝ Low^ b^	The evidence suggests no differences in the perceptions of pain in individuals treated with an open or closed technique using a golden chain
Patient’s perception of pain	15 cases, 11 controls (1 observational study)	⨁⊝⊝⊝ Very low ^c^	The evidence suggests no differences in the perceptions of pain in individuals treated with either miniscrews or transpalatal arch using a cantilever spring
Root resorption	15 cases, 11 controls (1 observational study)	⨁⊝⊝⊝ Very low^ c^	There was a significant difference in the volume of lateral incisor root resorption between the miniscrews group and the transpalatal arch group

**Table 9:** Summary of findings according to the GRADE guidelines for the included studies * The risk in the intervention group (and its 95% CI) is based on the assumed risk in the comparison group and the relative effect of the intervention (and its 95% CI). ^a^ Decline two levels for risk of bias in [31] (unclear risk of bias of randomization process, unclear risk of bias in the measurement of outcomes), and one level for imprecision**. ^b^ Decline two levels for risk of bias in [43] (unclear risk of bias of deviation from intended intervention, unclear risk of bias in the measurement of outcomes), and one level for imprecision**. ** Limited number of trials and limited sample size. GRADE: Grading of Recommendations Assessment, Development, and Evaluation; RCT: randomized controlled trial.

Patient or population: patients with palatally impacted canines; Intervention: conventional technique of canine retraction; Comparison: another technique of canine retraction without acceleration			
Outcomes	No. of participants (studies)	Certainty of the evidence (GRADE)	Comments
Duration of complete orthodontic treatment	12 (1 RCT)	⨁⊝⊝⊝ Very low ^a^	There was a significant difference in the duration of complete orthodontic treatment between the acceleration and the conventional groups
The velocity of impacted canine movement	12 (1 RCT)	⨁⊝⊝⊝ Very low ^a^	The evidence suggests that the acceleration technique results in a greater canine movement rate compared to the conventional technique
The velocity of impacted canine movement	12 (1 RCT)	⨁⊝⊝⊝ Very low ^b^	There was a significant difference in the rate of impacted canine movement between the intervention group and the control group
Periodontal outcomes	12 (1 RCT)	⨁⊝⊝⊝ Very low^ a^	There were no clinical differences between the corticotomy-assisted canines and their contralateral teeth regarding the periodontal probing and bone levels
Periodontal outcomes	12 (1 RCT)	⨁⊝⊝⊝ Very low ^b^	There was no statistical difference between the two study groups in terms of the width of keratinized gingiva and gingival recession variables, while a statistical difference was found between the two groups in the alveolar bone thickness

Discussion

To the best of our knowledge, this systematic review is the first to evaluate the effectiveness of surgical and non-surgical methods in accelerating PIC movement. In addition, it seems to be that this is the first systematic review to assess the efficacy of different mechanical traction methods when moving and aligning PICs in terms of speed, periodontal status, and patient-reported outcomes.

Risk of Bias of Included Studies

In this systematic review, six out of the eight RCTs and one CCT were judged to be at high risk of bias. This may lead to low confidence in the results and their ability to represent true treatment effects. As a result, these studies provided a low level of evidence and precluded the quantitative synthesis of results in a meta-analysis. Two studies out of eight were judged to be at unclear risk of bias, and this could have partially affected obtaining robust conclusions.

Velocity and Duration of Orthodontic Traction/Treatment

Conventional technique versus another conventional technique: The two studies of Smailiene et al. and Parkin et al. [7,41], which presented a low risk of bias in considering eruption duration outcome, stated that the open exposure technique resulted in more reduction in the time needed for impacted canine's traction. There was a mean difference of about seven months between the corresponding intervention groups in both studies regarding the duration of the canine eruption. This can be attributed to the differences in the initial sample's characteristics, in terms of initial depths of impaction, impacted canine angulation, participant's age, and the differences in the mechanical traction techniques. Smailiene et al. [7] compared the use of the ballista loop (with the closed flap surgery) with the open surgical approach and free eruption, while Parkin et al. [41] used the power chain and the twin-wire technique for impacted canine's traction in their interventional groups. However, the authors of these two articles did not explain whether the obtained results were due to the mechanical traction techniques used (ballista loop, power chain, and the twin-wire) or were a result of the differences in the surgical intervention used (open or closed exposure), especially regarding the duration of orthodontic traction. According to the results of these two studies, it seems that the use of the power chain and the twin-wire may lead to better treatment results than the ballista loop. However, the strength of evidence in this context ranged from very low to low.

On the other hand, Heravi et al. [15] indicated that there was no significant difference in the duration of the canine eruption when using different mechanical anchorage methods with the same surgical exposure and mechanical traction methods (p = 0.125). In that study, which presented a high risk of bias, the forced eruption of the PICs in the experimental group was performed using a cantilever spring (made of 0.017 x 0.025-in titanium molybdenum alloy (TMA) wire) inserted into the slot of two miniscrews before the placement of brackets. While canine's dis-impaction in the control group was performed using a cantilever spring (made of 0.016 x 0.022-in stainless steel) soldered to the palatal bar after initial leveling and alignment. The erupted canines were guided to the arch with the aid of NiTi overlay wires. However, using miniscrews in the direct anchorage (i.e. by connecting the traction force directly to the miniscrews) seemed not to reduce the treatment duration significantly, but it lead to better treatment results. The strength of evidence was very low.

Regarding the complete orthodontic treatment duration, the results of several studies indicated that the average duration of treatment for PICs was between 18 and 30 months [7,43]. The study by Smailiene et al. [7] stated that the open exposure technique resulted in more reduction in the complete orthodontic treatment duration. They indicated that the patient's age (at the start of treatment) and the initial localization (horizontal and vertical) of the impacted canine were not associated with the treatment duration. According to GRADE, the strength of evidence was very low.

An accelerated technique versus a conventional one: The study of Fischer [31], which presented a high risk of bias, stated that the corticotomy-assisted technique resulted in more reduction in the complete orthodontic treatment duration than the conventional technique. This could be explained by the RAP taking place in the involved area, which is characterized by an increase in cortical bone porosity due to increased osteoclastic activity following surgical wounding of cortical bone, which in turn presented less resistance to impacted canines movement [44,45]. However, the sample size was so small; therefore, the conclusions related to this study were of a very low level of evidence.

Fischer [31] and Dehis et al. [43] stated that the acceleration techniques resulted in increasing the velocity of PIC's traction movement. When comparing the results of these two studies, a greater increase in the rate of impacted canine movement was reported when the vitamin C injection procedure was used. This can be attributed to the frequent use of vitamin C injections (every two weeks), compared with the corticotomy procedure performed only once during surgical exposure in the other study [31]. Therefore, future research work should consider evaluating the effect of repeated application of the surgical methods at intervals between two and three months due to the temporary nature of the RAP and long-term procedure with a path of forced eruption that may take up to 10 mm of distance. Thus, repeated application of the surgical technique may lead to continuous activation of the RAP throughout the whole course of traction [45,46]. The high heterogeneity between the two studies could not allow for a quantitative synthesis of the findings. The strength of evidence regarding this point ranged from very low to low.

Periodontal Outcomes

Conventional technique versus another conventional technique: Regarding the periodontal outcomes, non-significant differences between the experimental and control groups were reported in four articles [7,15,41,42]. These studies used different mechanical traction methods and reported different periodontal outcomes so the high heterogeneity between the studies could not allow for quantitative synthesis of the findings. Smailiene et al. [7,42] assessed the periodontal pocket depth (PPD) on the side of the previously impacted canine and found that it did not exceed 3 mm (mean depth = 2.2 mm; SD: 0.55), whereas, on the contralateral side, the mean depth was 2.01 mm (SD: 0.42; p < 0.05). They claimed that the localization of deeper periodontal pockets could depend on the initial localization of the impacted canines and on the treatment mechanics. However, no information about the effect of the ballista loop, used as a mechanical traction method in these two studies, on the PPD was given and no comparison was made between this mechanical method and the other ones.

On the other hand, Parkin et al. [41] evaluated the clinical periodontal attachment level (CAL= periodontal probing depth + gingival recession), and no statistically significant difference was found between the study groups, whereas a statistical difference in mean attachment loss was found between operated and unoperated (contra-lateral) canines. They concluded that exposure and alignment of PDCs (by moving the impacted canines above or below the mucosa) have a small impact on periodontal health. However, this study did not give any evidence of the effect of the mechanical technique used for traction (twin wire or elastic chain) on clinical attachment loss.

Two of the included trials [7,41] evaluated the gingival recession status. Smailiene et al. [7] reported that the differences in gingival recession between groups and between the test and contralateral sides were nonsignificant (the mean amount of recession ranged between 0 and 0.5 mm). In contrast, Parkin et al. [41] reported a statistically significant difference in the gingival recession between operated and unoperated canines, while the differences between groups were nonsignificant (the mean amount of recession ranged between 0 and 2 mm). The differences between the two studies can be explained by the difference in sample size, measurement areas, measurement method, follow-up duration, and probably the difference in the mechanical traction method.

Two of the included trials [7,41] reported bone support. Smailiene et al. [7] reported that the differences between the test groups were nonsignificant, but in comparison with the contralateral side, significant differences were found on the mesial side of the canine and the distal side of the lateral incisor. Parkin et al. [41] reported a non-significant difference between open and closed groups (p = 0.936) and a statistically significant difference between operated and unoperated canines. However, the authors of these two articles relied on a two-dimensional radiographic evaluation, which does not give a complete picture of the amount of bony support around the extruded canines. Therefore, future research work should take into account the three-dimensional radiographic evaluation of the erupted canine bone support, which can give a more accurate assessment of this outcome.

One study [7] reported the width of KT on the impacted and contralateral canine. They concluded that the width of KT was greater in the open technique group, but the differences in measurements between groups and between the test and contralateral sides were nonsignificant. More studies should evaluate this variable, especially its relationship with the type of surgical exposure, the type of surgical sutures, the type of mechanical traction, and the possible effects when using a surgical acceleratory method from the buccal side.

Heravi et al. [15] assessed the GI and BOP index and found no statistically significant differences between the miniscrews group and the TPA group regarding these two variables. However, the other important periodontal outcomes were not evaluated, so the overall perception of the periodontal statement was not completely reliable. The strength of evidence regarding this context ranged from very low to low.

An accelerated technique versus a conventional one: The included trials [31,43] reported that there were no significant differences in the periodontal status between the acceleration and conventional groups, except for a significant difference in the alveolar bone thickness (p = 0.002) that was found between the intervention groups (locally injected vitamin C group versus conventional traction group) in the study of Dehis et al. [43]. Fischer evaluated the periodontal probing depth and bone levels. Whereas Dehis et al. assessed the amount of keratinized gingiva, the gingival recession, and the alveolar bone thickness. However, these two studies lacked the assessment of other important periodontal outcomes. Therefore, future research work should cover all periodontal outcomes. The strength of evidence for these outcomes ranged from very low to low.

Patient-Reported Outcomes

No significant differences in the level of pain and discomfort were reported between the interventional groups in three out of the included studies [12,15,17], whereas Björksved et al. [40] reported that patients experienced significantly more post-surgery pain and impairment in the open surgical technique group than in the closed surgical technique group. This can be explained by the use of glass ionomer cement on the exposed canine crown to prevent gingival overgrowth during the spontaneous eruption in the study of Björksved et al., while both Gharaibeh and Al-Nimri and Parkin et al. sutured a pack in the exposed operation in the open exposure groups. In addition, in Björksved et al.'s trial, patients were not allowed to freely take painkillers, unlike in those in the studies of Gharaibeh and Al-Nimri and Parkin et al., and this could have affected the obtained results.

Gharaibeh and Al-Nimri [12] reported that pain scores were greater in the closed-eruption group (33% in the closed-eruption compared with 22% in the open-eruption group) on the first postoperative day, but the pain regression was faster in the closed-eruption group. Parkin et al. [17] reported that pain was evident in the immediate postoperative period, lasted for a short time, and subsided after a few days. However, the authors did not report the effect of their use of gold chains as a means of traction on pain and discomfort levels. The high heterogeneity between the previous studies [12,17] did not allow for a quantitative synthesis of the findings.

In the study of Heravi et al. [15], pain experience was measured three weeks after initial loading and at the end of disimpaction treatment. Higher pain levels were detected in the control group than in the experimental group after three weeks but, at the end of treatment, this difference was not statistically significant (p = 0.769). This can be explained by the difference in the intensity of the mechanical force used in traction due to the difference in the material of which the spring was made between the two study groups. However, the author did not indicate that the use of a cantilever spring affected this result and only indicated that the use of temporary anchorage devices (TADs) did not cause pain and discomfort during placement and treatment. The strength of evidence in this context ranged from very low to low.

Root Resorption

Heravi et al. [15] reported that no statistically significant differences were found between the two study groups regarding the volume of canine root resorption. On the other hand, greater lateral incisors root resorption was detected in the control group than in the experimental group. The researchers attributed these differences to the use of TADs that allowed more controlled movement of the impacted canines away from the roots of adjacent teeth before a comprehensive arch orthodontic setup. However, the authors did not mention the advantages of using the cantilever springs as a means of impacted canine traction over other mechanical means. The strength of evidence was very low.

Limitations of the current review

One of the limitations of the current review is being based only on English-written papers. Eight RCTs and one CCT were found and included in this systematic review, but the strength of evidence ranged from "low" to "very low." The high heterogeneity among the retrieved studies, the differences in the applied interventions, studies' designs, studied outcomes and patient ages, the variability in the methods of outcome evaluation, and the variability in the biomechanics used for canine traction between the included trials did not allow for conducting a quantitative synthesis of the findings.

## Conclusions

Combining the open surgical technique with some mechanical means (such as a power chain, ballista loop, and twin-wire or cantilever spring directly anchored by miniscrews) can reduce the orthodontic treatment duration of the PICs when conventional non-accelerated traction methods are used. However, the strength of evidence in this regard is low to very low. The use of direct anchorage by miniscrews to move the PICs away from the adjacent teeth roots before the alignment of teeth can lead to a reduction in root resorption. The strength of evidence in this regard is "very low." There were no significant differences in the periodontal outcomes between intervention groups when different mechanical means were used for conventional PICs' traction, whether open or closed traction technique was used, while significant differences existed between operated and un-operated canines. However, the strength of evidence in this regard is low to very low.

Orthodontic traction of PICs by conventional methods was generally associated with mild to moderate pain levels lasting several days in the short-term follow-up (up to 10 days). The severe pain on the first postoperative day subsided faster during the following days when using the closed traction technique. However, contradictory results were found regarding the severity of the perceived pain when comparing the open and closed traction methods. The relationship between the pain/discomfort levels and the mechanical traction method was not evaluated in the retrieved studies. The use of accelerated methods for PICs' traction can lead to an increase in the velocity of traction movement with no significant differences in periodontal outcomes between accelerated and conventional methods. However, the evidence supporting these findings is weak to very weak. The quality of evidence ranged from very low to low, concerning velocity, duration of orthodontic traction/treatment, patient-reported outcomes, and periodontal variables. Therefore, further randomized controlled trials should be conducted to allow quantitative data synthesis. We also recommend performing further studies to evaluate the efficacy of using orthodontic acceleration techniques in speeding up the traction of impacted canines such as piezocision, cortico-alveolar perforations, corticision, and low-level laser therapy.
